# Editorial: Antivirulence Drugs Against Bacterial Infections

**DOI:** 10.3389/fmicb.2021.690672

**Published:** 2021-05-25

**Authors:** Francesco Imperi, Wangxue Chen, Younes Smani

**Affiliations:** ^1^Department of Science, Roma Tre University, Rome, Italy; ^2^Human Health Therapeutics Research Center (HHT), National Research Council Canada, Ottawa, ON, Canada; ^3^Department of Biology, Brock University, St. Catharines, ON, Canada; ^4^Clinical Unit of Infectious Diseases, Microbiology and Preventive Medicine, University Hospital Virgen del Rocío, Seville, Spain; ^5^Institute of Biomedicine of Seville (IBiS), University Hospital Virgen del Rocío/Spanish National Research Council (CSIC)/University of Seville, Seville, Spain

**Keywords:** antivirulence, drug, infection, treatment, bacteria

Antimicrobial resistance poses a well-recognized public health threat worldwide due to the global dissemination of bacteria resistant to multiple antibiotic classes and the development of severe infections such as bacteremia, pneumonia, urinary tract, and intraabdominal infections. Finding solutions to fight against antibiotic-resistant bacterial infections is deemed a global priority by the World Health Organization and other international institutions. Few novel antibiotic families are under clinical evaluation, and those in the most advanced stage of development are being developed against Gram-positive bacteria. For example, Ridinilazole, an inhibitor of cell division and toxin production, is under phase 3 clinical trial for treatment of *Clostridium difficile* infections. Afabicin, an inhibitor of FabI (NAD(P)H)-dependent trans-2-enoyl-ACP reductase, is under phase 2 clinical trial for treatment of acute skin, bone, and joint infections by *Staphylococcus aureus*. Regarding antibacterials active against Gram-negatives, GSK3882347, a FimH antagonist developed for treatment of urinary tract infection by *Escherichia coli*, and RC-01, an LpxC inhibitor developed for treatment of severe infections by Gram-negative bacilli, are under phase 1 clinical trials. Meanwhile POL7080, a peptidomimetic targeting the lipopolysaccharide transport protein LptD, has successfully completed a phase 2 trial in patients with *Pseudomonas aeruginosa* lung infections. But its phase 3 clinical trial development has been stopped due to renal failure in patients. The need to increase the arsenal of antimicrobial agents warrants the exploration of new antimicrobial therapeutic strategies for use alone or in combination with currently available antibiotics. In this environment, drugs that specifically target bacterial virulence factors involved in the bacterial pathogenesis such as outer membrane proteins, toxins, siderophores, and secretion systems, etc., rather than cell viability have received considerable attention. Such an approach is less likely to induce selective pressure for resistance on pathogenic bacteria and may provide novel solutions against the emergence and spread of antimicrobial resistance. Despite this attractiveness, only a few antivirulence therapeutics are currently in clinical development.

In this Research Topic focused on antivirulence strategies against bacterial infections, seven works (six original research articles and one review) were published on the use of peptides, antibiotics, and natural products as antivirulence compounds.

In reference to the use of peptides, two approaches (tryptophan-containing linear antimicrobial peptides and tryptophan-containing cyclic hexapeptides) were analyzed in monotherapy and in combination with antimicrobial agents against Gram-negative pathogenic bacteria. Shang et al. analyzed the effects of a series of tryptophan-containing peptides (TCP) on quorum sensing-regulated virulence and biofilm development of multidrug-resistant *P. aeruginosa*. They found that TCP at low μM concentrations (corresponding to 1/2-1/8 MIC) exhibited significant reduction of quorum sensing and biofilm formation associated with the inhibition of extracellular polysaccharide production by downregulating transcription of exopolysaccharide biosynthetic genes. In addition, they showed that combination of TCP with ceftazidime and piperacillin provides synergistic effects against multidrug-resistant *P. aeruginosa*.

Ayerbe-Algaba et al. performed an *in vitro* screening of a library of derivatives of the cyclic-hexapeptide AOA-2 that was previously demonstrated to inhibit the outer membrane protein A (OmpA) of Gram-negative bacilli. They found that two derivatives RW01 (addition of NH_2_ to increasing of AOA-2 solubility) and RW06 (substitution of arginine in AOA-2 by its analog 2,4-diaminobutanoic acid) at 31.25 and 62.5 μg/mL have increased ability to reduce the interaction of *Acinetobacter baumannii, P. aeruginosa* and *E. coli* to human lung epithelial cells when compared to the lead compound. Moreover, RW01 and RW06 potentiated the activity of colistin against *A. baumannii* at levels comparable to AOA-2.

In addition, the antibacterial activity of two natural products were studied against Gram-positive bacteria such as *S. aureus*. The first one, eriodictyol, which is an antioxidant flavonoid present in citrus fruits, was shown to attenuate the virulence of *S. aureus in vitro* and in a murine model of pneumonia at 64 μg/mL and 100 mg/kg, respectively. Mechanistic analysis showed that eriodictyol inhibits the production of sortase A (SrtA), a membrane-bound cysteine transpeptidase involved in the catalysis of covalent anchoring of surface proteins to the cell wall (Wang et al.). The second natural product described in this Research Topic is a glycosylated paeoniflorin-like compound (PRAE-a) extracted from the Chinese herb *Paeoniae radix*. PRAE-a at 1 mg/mL was found to inhibit the hemolytic activity of *S. aureus* α-toxin *in vitro*, likely by preventing its oligomerization to form the heptameric pore complex. When administered at 1 mg/g, PRAE-a also reduced α-toxin levels and *S. aureus* associated lung injury and lethality in a mouse pulmonary infection model (Liu et al.).

Kumar et al. further provided evidence that antibiotics at sub-inhibitory concentrations can affect quorum sensing in bacteria. Using *P. aeruginosa* as the model system, the authors showed that sub-MIC of cephalosporins (cefepime, ceftazidime, and ceftriaxone) not only significantly inhibited the motility, virulence factor (pyocyanin) production, and biofilm formation, at levels comparable to those of quorum sensing-defective mutants, but also exhibited synergy with aminoglycoside antibiotics. Molecular docking studies predicted the interaction of cephalosporins with the LasR and PqsR quorum sensing receptors. Finally, infection assays proved that sub-inhibitory concentrations of cephalosporins drastically reduced *P. aeruginosa* pathogenicity in the *Caenorhabditis elegans* model.

An approach based on nano-micelles that contain iodine (FS1) was also investigated in this Research Topic. Reva et al. examined the effect of FS1 on the gene expression and epigenetic modifications, important for the virulence and antimicrobial resistance of *S. aureus* and *E. coli*. The study demonstrated that treatment with FS1 (450 μg/mL) induced: (i) a profound transition of the metabolism of *S. aureus* toward anaerobiosis due to damaging of oxygen-dependent terminal cytochrome, (ii) an increase in the rate of mutations in *S. aureus* due to oxidation and/or halogenation of chromosomal DNA nucleotides that eventually may affect many important cellular systems including antibiotic resistance genes; (iii) osmotic, oxidative and carbonyl stresses which may aggravate the antibiotic resistance fitness cost; and (iv) destabilization and loss of horizontally acquired antibiotic resistance genomic islands (e.g., SCCmec) and virulence plasmids in *E. coli*.

Finally, Ford et al. reviewed recent studies on antivirulence approaches against *S. aureus*, with a particular focus on strategies targeting pore-forming toxins, immune evasion mechanisms, and quorum sensing, as the research on these antivirulence targets provides broad principles that can apply to different bacterial pathogens. The authors also outlined and critically discussed major challenges of antivirulence research, such as the potential for resistance development and the need for reliable animal models to select lead compounds that should advance to clinical trials.

In conclusion, antivirulence drug development has gained interest and momentum in the last decade, as reflected in the works published in this special issue of Frontiers in Microbiology, most of them performed *in vitro*. Additional relevant issues should be considered for the pre-clinical development of such compounds, including the use of animal models of infections for the assessment of their therapeutic efficacy, the determination of pharmacokinetic parameters, and the achievement of safety studies. The preclinical and clinical development of these approaches may help in the future to increase the arsenal of antibacterial drug families that can be used in monotherapy and/or in combination with antibiotics for the treatment of multidrug resistant bacterial infections.

## Author Contributions

YS and FI drafted the manuscript. All authors reviewed and approved the final manuscript.

## Conflict of Interest

The authors declare that the research was conducted in the absence of any commercial or financial relationships that could be construed as a potential conflict of interest.

